# Second-Generation Long-Acting Injectable Antipsychotics and the Risk of Treatment Failure in a Population-Based Cohort

**DOI:** 10.3389/fphar.2022.879224

**Published:** 2022-05-19

**Authors:** Donica Janzen, James M. Bolton, Christine Leong, I fan Kuo, Silvia Alessi-Severini

**Affiliations:** ^1^ College of Pharmacy, Rady Faculty of Health Sciences, University of Manitoba, Winnipeg, MB, Canada; ^2^ Department of Psychiatry, Max Rady College of Medicine, Rady Faculty of Health Sciences, University of Manitoba, Winnipeg, MB, Canada; ^3^ Pharmaceutical, Laboratory and Blood Services Division, Ministry of Health, New Westminster, BC, Canada

**Keywords:** antipsychotic treatment, long-acting injectable and oral antipsychotics, real-world data, comparative effectiveness, psychotic disorders

## Abstract

**Introduction:** Second-generation long-acting injectable antipsychotics (SG-LAIAs) may improve outcomes compared to other antipsychotics. Real-world studies using linked administrative databases play an important role in assessing the comparative effectiveness of antipsychotic medications.

**Methods:** We used a prevalent new-user design in a population-based cohort of antipsychotic users with diagnosis of a psychotic disorder to compare the primary outcome of treatment failure, defined as psychiatric hospitalization, completed suicide, incarceration, or treatment discontinuation. Additional outcomes were all-cause mortality. SG-LAIA users were matched on a 1:1 basis with other antipsychotic users based on the time-conditional propensity score, calendar time, and prior antipsychotic exposure.

**Results:** The use of LAIAs was not associated with a lower risk of treatment failure than other antipsychotics (adjusted hazard ratio 1.07 and 95% confidence interval 0.98–1.15) but did reduce all-cause mortality (adjusted hazard ratio 0.69 and 95% confidence interval 0.48–0.99). Monotherapy with LAIAs was superior to other antipsychotic monotherapy (adjusted hazard ratio for treatment failure 0.83 and 95% confidence interval 0.78–0.89), and LAIAs were superior to other antipsychotics in antipsychotic-naïve users (adjusted hazard ratio for treatment failure 0.57 and 95% confidence interval 0.47–0.70).

**Conclusion:** In this population-based cohort, SG-LAIAs reduced the risk of treatment failure in incident new users but not in prevalent new users.

## Introduction

Long-acting injectable antipsychotics (LAIAs) have an established role for patients who require long-term antipsychotic treatment and are at risk of poor adherence; LAIAs improve adherence and persistence to antipsychotic treatment, which subsequently reduces the risk of relapse (Correll et al., 2016; Pilon et al., 2017; Greene et al., 2018). Clinical practice guidelines for the treatment of schizophrenia recommend that all patients should be presented with LAIAs as a treatment option (Remington et al., 2017; American Psychiatric Association, 2021). The availability of long-acting injectable formulations of second-generation antipsychotics may improve patient acceptance of LAIAs, but their increased cost and formulary restrictions in some jurisdictions may be a barrier to the widespread use of second-generation long-acting injectable antipsychotics (Kane et al., 2019).

Observational study designs or pragmatic trials may be preferred over randomized controlled trials in studies of long-acting injectable antipsychotic effectiveness as they are more inclusive of patients with histories of non-adherence to treatment and multiple comorbidities (Alphs et al., 2014; Tiihonen et al., 2017). Randomized clinical trials have produced conflicting results, with some showing a reduced risk of relapse and treatment failure with LAIAs (Alphs et al., 2015; Subotnik et al., 2015), while others found no significant difference compared to oral antipsychotics (Kishimoto et al., 2014; Buckley et al., 2016). Improved clinical outcomes resulting from greater adherence to LAIAs may be obscured in controlled trials where adherence to assigned treatment is closely monitored (Correll et al., 2016). Observational studies also have limitations, most notably persistent confounding by unmeasured variables. Well-conducted observational studies can mitigate the risk of confounding with the incident user, active comparator designs, the use of propensity scores, and adjusting for measured covariates (Ray, 2003; Stürmer et al., 2014; Lund, Richardson and Sturmer, 2015). However, incident user designs limit sample size, particularly in the case of SG-LAIAs, where most new users have switched from an alternate antipsychotic. Prevalent new-user designs allow for the comparison of “switchers” to a newly marketed medication without restricting to treatment-naïve users (Suissa, Moodie and Dell’Aniello, 2017; Filion et al., 2020).

Many studies evaluate antipsychotic effectiveness in terms of treatment failure, treatment discontinuation, and hospitalization, but other outcomes may also be meaningful in this patient population. Population-based studies have established that patients with psychotic disorders are at increased risk of criminal justice system involvement (Khalifeh et al., 2015; Dean et al., 2018; Sariaslan et al., 2020). Antipsychotics may prevent reoffending in individuals with a history of incarceration (Fazel et al., 2014; Alphs et al., 2015; Chang et al., 2016; Rezansoff et al., 2017), but the literature on the role of antipsychotics in reducing crime in individuals without a history of justice system involvement is lacking. In the present study, we have used a prevalent new-user design in a population-based cohort of antipsychotic users to evaluate the risk of treatment failure, a composite endpoint of psychiatric hospitalization, completed suicide, incarceration, and treatment discontinuation, in SG-LAIA users versus oral antipsychotic users.

## Materials and Methods

### Data Source

We used the Manitoba Population Research Data Repository, a collection of administrative health, education, social, justice, and registry databases, housed at the Manitoba Centre for Health Policy in Manitoba, Canada, to form a cohort of second-generation long-acting injectable antipsychotic (SG-LAIA) users (Suissa, Moodi,e and Dell’Aniello, 2017; Smith et al., 2018). The repository captures all prescriptions dispensed in the province of Manitoba, Canada, excluding in-hospital pharmaceuticals, and has been validated for SG-LAIAs (Janzen et al., 2022). We linked prescription claims to hospital discharge abstracts, medical service claims, prosecutions, vital statistics, and insurance registry data by a scrambled personal identification number. This study received ethics approval from the University of Manitoba Health Research Ethics Board under the project number HS20380 (H2016:468), the Manitoba Centre for Health Policy, the Health Information Privacy Committee, and Manitoba Justice.

### Cohort Selection and Exposure Definition

We formed a base cohort of all individuals who were dispensed antipsychotic medication on the first date. An SG-LAIA was dispensed in Manitoba between 14 February 2005 and 31 March 2020 and had ≥ 1 year of continuous registration in the Manitoba Health Services Insurance Plan and ≥ 1 medical or hospital claim with a diagnosis of a psychotic disorder in the 3 years prior to cohort entry (Chartier et al., 2018). From the base cohort, we formed prevalent and incident new-user cohorts. Prevalent new users were defined as individuals who were dispensed an SG-LAIA and had a previous antipsychotic prescription in the 1 year prior to the SG-LAIA dispensation but no prior SG-LAIA in the 1-year look-back window. Incident new users were defined as individuals who were dispensed a new SG-LAIA with no prior antipsychotic dispensation in the previous year. For SG-LAIA new users, the cohort entry date (t_0_) was defined as the date of the first dispensation. Subjects who received the oral equivalent of the incident SG-LAIA for less than 30 days before t_0_ were included in the incident new-user cohort. For each SG-LAIA new user, we created an exposure set of eligible comparators who were dispensed antipsychotic medication within 120 days of t_0_ and had the same prior duration of continuous antipsychotic use ± 180 days, prior year use of clozapine, prior year antipsychotic medications (0–1 or ≥ 2), and prior exposure to first-generation LAIA. For comparators, t_0_ was defined as the dispensation date of any dispensation included in an exposure set. Subjects were excluded if the cohort exit date occurred on t_0_. Additional subjects were excluded from the SG-LAIA new-user cohort if they had an incident antipsychotic dispensation other than an SG-LAIA on t_0_ or if there were no eligible comparators in their exposure set.

The cohort members were included in a monotherapy subgroup if they were dispensed only one antipsychotic medication on t_0_. Subjects in the monotherapy subgroup were censored upon the dispensation of an antipsychotic other than the incident antipsychotic.

### Propensity Score Matching

Within each exposure set, we determined the propensity for initiation of SG-LAIAs at t_0_. For comparators, a time-conditional propensity score was calculated at each antipsychotic dispensation date in an exposure set. Covariates included in the propensity score were sex, age, income quintile, number of prior year medication classes dispensed, number of prior year hospitalizations, number of prior year physician visits, time since psychotic disorder diagnosis (defined as earliest of first antipsychotic dispensation or first hospitalization or medical claim with a diagnosis of psychotic disorder), prior year dispensation of psychotropic medication, psychiatric diagnoses in the previous 3 years, being accused of a crime in the previous 3 years, being a victim of a crime in the previous 3 years, and the calendar year of t_0_. Exposure sets were excluded if the propensity score of the SG-LAIA new user was outside the range of propensity scores of comparators in the exposure set. SG-LAIA new users were matched on the basis of 1:1 with replacement with the comparator in the exposure set with the nearest time-conditional propensity score. Matching was performed in the chronological order, starting with the subject with the earliest t_0_.

### Outcome Definition

The cohort members were followed from t_0_ to the occurrence of the outcome, death, emigration from Manitoba, or 31 March 2020. In addition, comparators were censored if they received SG-LAIA dispensation. The primary outcome was treatment failure, defined as psychiatric hospitalization (including hospitalization for a mood/anxiety disorder, substance use disorder, psychotic disorder, schizophrenia, or attempted suicide), incarceration, suicide (the primary cause of death being self-inflicted injury or poisoning or poisoning of undetermined intent), or treatment discontinuation (defined as a gap in prescription dispensations greater than 90 days). Additional outcomes included all-cause mortality and individual components of the composite primary outcome. We also conducted a subgroup analysis of prevalent and incident new users and restricted to subjects exposed to antipsychotic monotherapy only during the follow-up. Detailed definitions of outcomes are provided in Supplementary Table S1.

### Statistical Analysis

We used descriptive statistics to evaluate cohort characteristics. We determined standardized differences to assess the covariate balance between exposure groups before and after matching. Outcomes were analyzed using a Cox proportional hazards regression model stratified by matched pair, adjusting for age, sex, time since psychotic disorder diagnosis, decile of the time-conditional propensity score, prior year hospital admissions, history of being accused of a crime, and diagnosis of personality disorder, substance use disorder, or mood/anxiety disorder. A robust sandwich variance estimate was included in the Cox model to account for matching with replacement. In addition, we used a modified Cox model to perform adjustments for the time-varying use of antipsychotic polypharmacy during the follow-up. All analyses were conducted in SAS® 9.4 (SAS Institute; Cary, NC).

### Sensitivity Analysis

We repeated analyses in cohort members who had received a diagnosis of schizophrenia (ICD-9-CM code 295 or ICD-10-CA code F20) in the 3 years prior to t_0_. We also conducted a *post hoc* sensitivity analysis including prior antipsychotics in the propensity score to evaluate the impact of baseline imbalance in prior antipsychotic medication.

## Results

### Description of the Cohort

**FIGURE 1:**
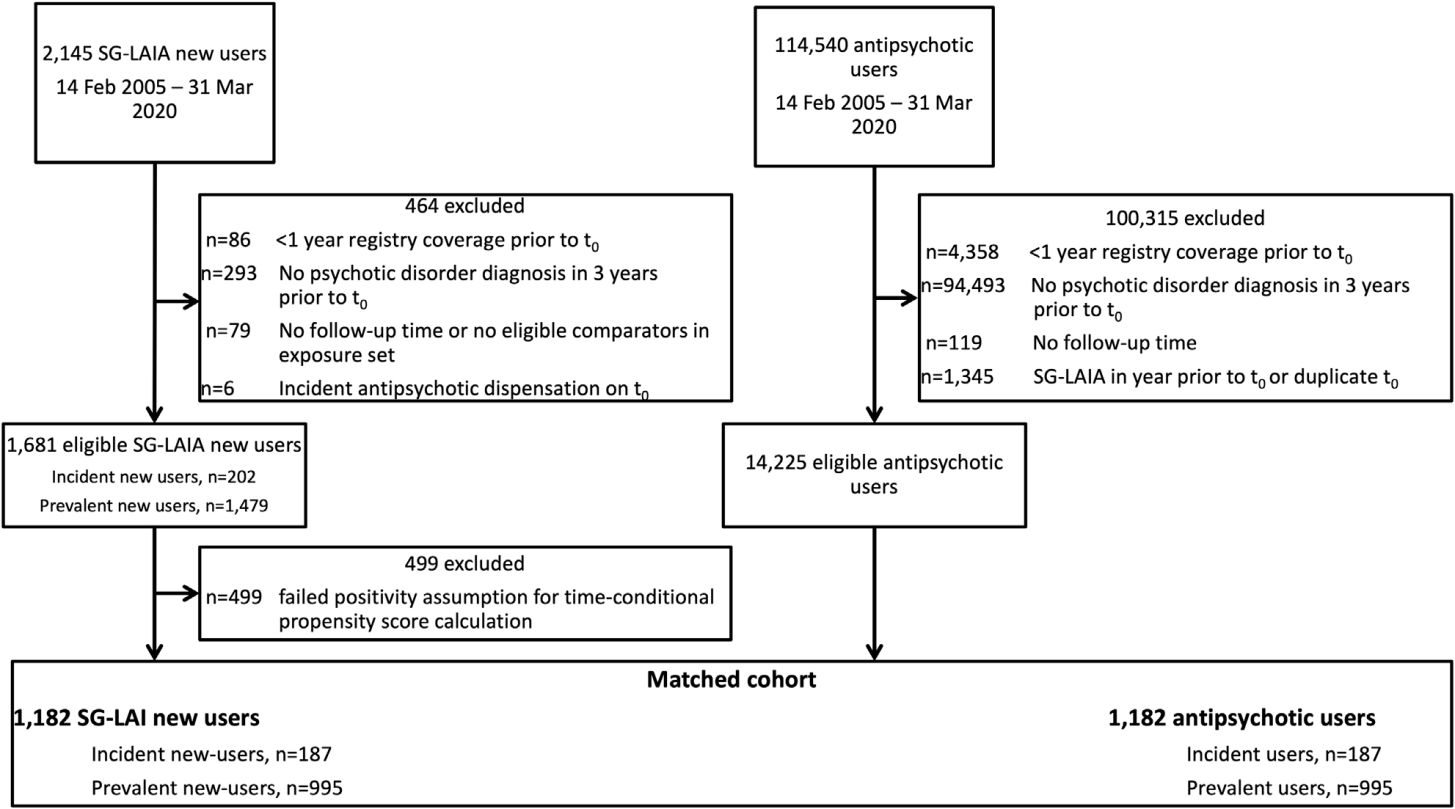
Cohort selection.

We identified 1,681 SG-LAIA new users and 14,225 antipsychotic user comparators eligible for matching. The final matched cohort included 1,182 matched pairs, with 187 in the incident new user cohort and 995 in the prevalent new-user cohort (Figure 1). The majority of SG-LAIA new users received risperidone-LAI (49.7%) on t0, followed by paliperidone-LAI (38.5%) and aripiprazole-LAI (11.8%). Among matched comparators, 86.9% received an oral SGA and 5.8% received an FG-LAIA on t0 (Supplementary Table S2). Baseline characteristics were well-balanced after matching, with standardized differences of less than 0.1 for all variables except for age groups less than 18 years and 18–30 years (Table 1, Supplementary Table S3). We adjusted Cox models for age to account for this imbalance.

**TABLE 1:** Baseline characteristics of cohort members before and after matching.

Characteristic	Before matching					After matching				
	SG-LAIA new users, *n* = 1,681		Antipsychotic users, *n* = 1,681^a^		Standardized difference	SG-LAIA new users, *n* = 1,182		Antipsychotic users, *n* = 1,182		Standardized difference
	n or mean	% or SD	n or mean	% or SD		n or mean	% or SD	n or mean	% or SD	
Females	604	35.9%	729	43.4%	0.15	435	36.8%	466	39.4%	0.05
Age (years)	36	16.3	50	21.0	0.72	37	17.2	37	17.4	0.00
Age group (years)										
<18	50	3.0%	70	4.2%	0.06	38	3.2%	111	9.4%	0.26
18–30	757	45.0%	314	18.7%	0.59	524	44.3%	424	35.9%	0.17
31–40	322	19.2%	221	13.1%	0.16	216	18.3%	237	20.1%	0.05
41–50	218	13.0%	279	16.6%	0.10	148	12.5%	178	15.1%	0.07
51–60	177	10.5%	300	17.8%	0.21	129	10.9%	120	10.2%	0.02
61–70	90	5.4%	198	11.8%	0.23	65	5.5%	51	4.3%	0.05
71–80	36	2.1%	113	6.7%	0.22	34	2.9%	23	1.9%	0.06
>80	31	1.8%	186	11.1%	0.38	28	2.4%	38	3.2%	0.05
Income quintile										
1 (lowest)	622	37.0%	597	35.5%	0.03	449	38.0%	457	38.7%	0.01
2	346	20.6%	369	22.0%	0.03	252	21.3%	241	20.4%	0.02
3	214	12.7%	232	13.8%	0.03	152	12.9%	143	12.1%	0.02
4	192	11.4%	169	10.1%	0.04	140	11.8%	145	12.3%	0.01
5 (highest)	127	7.6%	163	9.7%	0.08	93	7.9%	109	9.2%	0.05
Missing	180	10.7%	151	9.0%	0.06	96	8.1%	87	7.4%	0.03
Year of cohort entry										
2005/2006	45	2.7%	49	2.9%	0.01	41	3.5%	40	3.4%	0.00
2007/2008	81	4.8%	74	4.4%	0.02	60	5.1%	61	5.2%	0.00
2009/2010	105	6.2%	111	6.6%	0.01	74	6.3%	74	6.3%	0.00
2011/2012	183	10.9%	183	10.9%	0.00	124	10.5%	129	10.9%	0.01
2013/2014	196	11.7%	196	11.7%	0.00	129	10.9%	122	10.3%	0.02
2015/2016	363	21.6%	360	21.4%	0.00	246	20.8%	243	20.6%	0.01
2017/2018	413	24.6%	414	24.6%	0.00	288	24.4%	298	25.2%	0.03
2019/2020	295	17.5%	294	17.5%	0.00	224	19.0%	215	18.2%	0.02
Time since psychotic disorder diagnosis (years)	8.1	6.7	10.1	7.8	0.28	7.2	6.7	7.2	6.6	0.00
<1	237	14.1%	237	14.1%	0.00	229	19.4%	228	19.3%	0.00
1–4.9	499	29.7%	401	23.9%	0.13	375	31.7%	368	31.1%	0.01
5–10	341	20.3%	229	13.6%	0.18	197	16.7%	209	17.7%	0.03
>10	604	35.9%	814	48.4%	0.26	381	32.2%	377	31.9%	0.01
Prior FG-LAIA use	389	23.1%	389	23.1%	0.00	152	12.9%	152	12.9%	0.00
Prior year antipsychotic medications										
0–1	799	47.5%	799	47.5%	0.00	701	59.3%	701	59.3%	0.00
>1	749	44.6%	749	44.6%	0.00	407	34.4%	407	34.4%	0.00
Clozapine	133	7.9%	133	7.9%	0.00	74	6.3%	74	6.3%	0.00
Prior year number of medication classes dispensed										
0–1	454	27.0%	243	14.5%	0.31	338	28.6%	319	27.0%	0.04
2–5	604	35.9%	533	31.7%	0.09	432	36.5%	430	36.4%	0.00
>5	623	37.1%	905	53.8%	0.34	412	34.9%	433	36.6%	0.04
Prior year medication use										
Mood stabilizer	280	16.7%	309	18.4%	0.05	188	15.9%	169	14.3%	0.04
Antidepressant	595	35.4%	795	47.3%	0.24	414	35.0%	452	38.2%	0.07
Anxiolytic	612	36.4%	701	41.7%	0.11	403	34.1%	410	34.7%	0.01
Sedative-hypnotic	320	19.0%	412	24.5%	0.13	227	19.2%	225	19.0%	0.00
Anticonvulsant	94	5.6%	172	10.2%	0.17	70	5.9%	62	5.2%	0.03
Psychostimulant	51	3.0%	16	1.0%	0.15	33	2.8%	39	3.3%	0.03
Anticholinergic	397	23.6%	332	19.8%	0.09	233	19.7%	224	19.0%	0.02
Opioid	324	19.3%	371	22.1%	0.07	220	18.6%	222	18.8%	0.00
Opioid agonist therapy	13	2.2%	S	S	0.07	8	0.7%	14	1.2%	0.05
Smoking cessation aid	64	3.8%	68	4.0%	0.01	44	3.7%	41	3.5%	0.01
Alcohol use disorder drug	11	0.7%	S	S	0.06	S	S	S	S	0.03
Dementia drug	6	0.4%	65	3.9%	0.25	S	S	S	S	0.01
Antidiabetic drug	165	9.8%	278	16.5%	0.20	103	8.7%	108	9.1%	0.01
Antihyperlipidemic drug	130	7.7%	300	17.8%	0.31	86	7.3%	93	7.9%	0.02
Comorbidities										
Mood or anxiety disorder	1,283	76.3%	1,373	81.7%	0.13	906	76.6%	916	77.5%	0.02
Personality disorder	485	28.9%	472	28.1%	0.02	308	26.1%	308	26.1%	0.00
Substance use disorder	967	57.5%	726	43.2%	0.29	638	54.0%	632	53.5%	0.01
Dementia	201	12.0%	439	26.1%	0.37	146	12.4%	159	13.5%	0.03
Autism spectrum disorder	40	2.4%	41	2.4%	0.00	30	2.5%	29	2.5%	0.01
Intellectual disability/developmental disorder	199	11.8%	138	8.2%	0.12	117	9.9%	116	9.8%	0.00
ADHD	241	14.3%	120	7.1%	0.23	149	12.6%	171	14.5%	0.05
Suicide attempt	184	10.9%	144	8.6%	0.08	107	9.1%	120	10.2%	0.04
Prior year hospitalizations										
0	474	28.2%	1,064	63.3%	0.75	393	33.2%	391	33.1%	0.00
1–2	973	57.9%	550	32.7%	0.52	687	58.1%	690	58.4%	0.01
>2	234	13.9%	67	4.0%	0.35	102	8.6%	101	8.5%	0.00
Prior year physician visits										
0–2	61	3.6%	109	6.5%	0.13	54	4.6%	64	5.4%	0.04
3–5	127	7.6%	154	9.2%	0.06	97	8.2%	89	7.5%	0.03
>5	1,493	88.8%	1,418	84.4%	0.13	1,031	87.2%	1,029	87.1%	0.01
Incidents where accused of a crime										
0	1,156	68.8%	1,485	88.3%	0.49	851	72.0%	877	74.2%	0.05
1–2	232	13.8%	113	6.7%	0.23	153	12.9%	151	12.8%	0.01
>2	293	17.4%	83	4.9%	0.40	178	15.1%	154	13.0%	0.06
Incidents where victim of a crime										
0	1,585	94.3%	1,630	97.0%	0.13	1,129	95.5%	1,124	95.1%	0.02
>0	96	5.7%	51	3.0%	0.13	53	4.5%	58	4.9%	0.02

a Cohort before matching consisted of SG-LAIA new users and antipsychotic users in the exposure set of an SG-LAIA new-user design. Exposure sets were based on calendar time, prior duration of continuous antipsychotic use, prior year use of clozapine, prior use of FG-LAIA, and prior year number of unique antipsychotic medication dispensed. Characteristics of a random sample of one antipsychotic user per exposure set are reported. SG-LAIA users were matched on the basis of 1:1 with an antipsychotic user on the time-conditional propensity score.

ADHD = attention-deficit hyperactivity disorder; FGA = first-generation antipsychotic; FG-LAIA = first-generation long-acting injectable antipsychotic; LAI = long-acting injectable; S = suppressed due to count < 6; SD = standard deviation; SGA = second-generation antipsychotic; SG-LAIA = second-generation long-acting injectable antipsychotic.

The mean follow-up time for the primary outcome of treatment failure was 1.3 (SD 1.9) years for a total of 3,170 person years. In the SG-LAIA cohort, 913 experienced treatment failure in 1,512 person years of observation time for a crude incidence rate of 60.4 per 100 person years. Among matched comparators, there were 804 treatment failure events in 1,658 person years for a crude incidence rate of 48.5 per 100 person years. During 2,119 person years of observed monotherapy, there were 345 events in 653 person years of SG-LAIA monotherapy (crude incidence rate = 52.8 per 100 person years) and 704 events in 1,466 person years of monotherapy in the matched comparators (crude incidence rate = 48.0 per 100 person years). Baseline characteristics of the monotherapy subgroup are found in Supplementary Tables S4, S5.

### Results of Cox Models

The SG-LAIA use was not associated with a reduced risk of treatment failure compared to the matched antipsychotic users (adjusted hazard ratio 1.07 and 95% confidence interval 0.98–1.15) (Table 2). However, the risk of treatment failure was reduced during SG-LAIA monotherapy compared to matched antipsychotic monotherapy (adjusted hazard ratio 0.83 and 95% confidence interval 0.78–0.89) (Table 3). The SG-LAIA use had no impact on the risk of incarceration (adjusted hazard ratio 0.97 and 95% confidence interval 0.76–1.25) or treatment discontinuation (adjusted hazard ratio 1.00 and 95% confidence interval 0.91–1.09) but increased the risk of psychiatric hospitalization (adjusted hazard ratio 1.38 and 95% confidence interval 1.23–1.54). A small number of suicides were observed during the follow-up, so results are not reported. In addition, the SG-LAIA use reduced the risk of all-cause mortality in the overall cohort (adjusted hazard ratio 0.69 and 95% confidence interval 0.48–0.99) and during monotherapy (adjusted hazard ratio 0.10 and 95% confidence interval 0.02–0.44).

**TABLE 2:** Association between second-generation long-acting injectable antipsychotics versus oral antipsychotics and treatment failure, psychiatric hospitalization, incarceration, treatment discontinuation and all-cause mortality.

	Number of events	Person years	Crude incidence rate per 100 person years	Crude hazard ratio (95% CI)		Adjusted hazard ratio^a^ (95% CI)	
Treatment failure							
SG-LAIA new users	913	1,512	60.4	1.14	(1.10–1.17)	1.07	(0.98–1.15)
Matched antipsychotic users	804	1,658	48.5	1.00	(Reference)	1.00	(Reference)
Psychiatric hospitalization							
SG-LAIA new users	568	2,844	20.0	1.17	(1.11–1.25)	1.38	(1.23–1.54)
Matched antipsychotic users	484	2,979	16.2	1.00	(Reference)	1.00	(Reference)
Incarceration							
SG-LAIA new users	172	4,409	3.9	1.11	(0.97–1.27)	0.97	(0.76–1.25)
Matched antipsychotic users	155	3,965	3.9	1.00	(Reference)	1.00	(Reference)
Treatment discontinuation							
SG-LAIA new users	808	2,139	37.8	1.22	(1.17–1.27)	1.00	(0.91–1.09)
Matched antipsychotic users	663	2,157	30.7	1.00	(Reference)	1.00	(Reference)
All-cause mortality							
SG-LAIA new users	91	5,198	1.8	1.06	(0.88–1.27)	0.69	(0.48–0.99)
Matched antipsychotic users	86	4,552	1.9	1.00	(Reference)	1.00	(Reference)

a Adjusted for time-varying use of additional antipsychotic medication in each 3-month period of follow-up time and baseline variables that include age, sex, time since psychotic disorder diagnosis, decile of time-conditional propensity score, prior year hospital admissions, being accused of a crime, diagnosis of personality disorder, substance use disorder, and mood/anxiety disorder.

SG-LAIA, second-generation long-acting injectable antipsychotic.

**TABLE 3:** Association between second-generation long-acting injectable antipsychotic monotherapy versus oral antipsychotic monotherapy and treatment failure, psychiatric hospitalization, incarceration, treatment discontinuation, and all-cause mortality.

	Number of events	Person years	Crude incidence rate per 100 person years	Crude hazard ratio (95% CI)		Adjusted hazard ratio^a^ (95% CI)	
Treatment failure							
SG-LAIA new users	345	653	52.8	0.84	(0.79–0.90)	0.83	(0.78–0.89)
Matched antipsychotic users	704	1,466	48.0	1.00	(Reference)	1.00	(Reference)
Psychiatric hospitalization							
SG-LAIA new users	209	964	21.7	1.01	(0.86–1.18)	1.03	(0.86–1.24)
Matched antipsychotic users	373	2,215	16.8	1.00	(Reference)	1.00	(Reference)
Incarceration							
SG-LAIA new users	77	1,209	6.4	0.86	(0.67–1.10)	0.68	(0.43–1.09)
Matched antipsychotic users	125	2,797	4.5	1.00	(Reference)	1.00	(Reference)
Treatment discontinuation							
SG-LAIA new users	223	817	27.3	0.70	(0.60–0.82)	0.67	(0.57–0.79)
Matched antipsychotic users	485	1,697	28.6	1.00	(Reference)	1.00	(Reference)
All-cause mortality							
SG-LAIA new users	21	1,386	1.5	0.55	(0.34–0.86)	0.10	(0.02–0.44)
Matched antipsychotic users	63	3,312	1.9	1.00	(Reference)	1.00	(Reference)

a Adjusted for baseline variables age, sex, time since psychotic disorder diagnosis, decile of time-conditional propensity score, prior year hospital admissions, being accused of a crime, diagnosis of personality disorder, substance use disorder, and mood/anxiety disorder.

SG-LAIA, second-generation long-acting injectable antipsychotic.

### Subgroups

Notable differences were observed in prevalent new users compared with incident new users (Table 4). Prevalent new users of SG-LAIAs had an increased risk of treatment failure (adjusted hazard ratio 1.20 and 95% confidence interval 1.01–1.32), psychiatric hospitalization (adjusted hazard ratio 1.50 and 95% confidence interval 1.31–1.71), and treatment discontinuation (adjusted hazard ratio 1.13 and 95% confidence interval 1.03–1.25). In contrast, a reduced risk of treatment failure (adjusted hazard ratio 0.57 and 95% confidence interval 0.47–0.70), incarceration (adjusted hazard ratio 0.32 and 95% confidence interval 0.11–0.99), and treatment discontinuation (adjusted hazard ratio 0.52 and 95% confidence interval 0.40–0.66) was observed in incident new users.

**TABLE 4:** Association between second-generation long-acting injectable antipsychotics versus oral antipsychotics and treatment failure, psychiatric hospitalization, incarceration, treatment discontinuation, and all-cause mortality in prevalent and incident new users.

	Number of events	Person years	Crude incidence rate per 100 person years	Crude hazard ratio (95% CI)		Adjusted hazard ratio^a^ (95% CI)	
Treatment failure							
SG-LAIA prevalent new users	771	1,276	60.4	1.19	(1.15–1.23)	1.20	(1.10–1.32)
Matched antipsychotic prevalent users	648	1,541	42.1	1.00	(Reference)	1.00	(Reference)
SG-LAIA incident new users	142	236	60.2	0.91	(0.85–0.98)	0.57	(0.47–0.70)
Matched antipsychotic incident users	156	117	133.3	1.00	(Reference)	1.00	(Reference)
Psychiatric hospitalization							
SG-LAIA prevalent new users	480	2,384	20.1	1.19	(1.11–1.26)	1.50	(1.31–1.71)
Matched Antipsychotic prevalent Users	405	2,577	15.7	1.00	(Reference)	1.00	(Reference)
SG-LAIA incident new users	88	459	19.2	1.11	(0.96–1.29)	1.06	(0.81–1.30)
Matched antipsychotic incident users	79	402	19.7	1.00	(Reference)	1.00	(Reference)
Incarceration							
SG-LAIA prevalent new users	147	3,744	3.9	1.24	(1.06–1.44)	1.10	(0.85–1.43)
Matched antipsychotic prevalent users	119	3,425	3.5	1.00	(Reference)	1.00	(Reference)
SG-LAIA incident new users	25	665	3.8	0.69	(0.50–0.97)	0.32	(0.11–0.99)
Matched antipsychotic incident users	36	540	6.7	1.00	(Reference)	1.00	(Reference)
Treatment discontinuation							
SG-LAIA prevalent new users	685	1,829	37.5	1.31	(1.25–1.37)	1.13	(1.03–1.25)
Matched antipsychotic prevalent users	525	1,976	26.6	1.00	(Reference)	1.00	(Reference)
SG-LAIA incident new users	123	310	39.7	0.89	(0.81–0.98)	0.52	(0.40–0.66)
Matched antipsychotic incident users	138	181	76.2	1.00	(Reference)	1.00	(Reference)

a Adjusted for the time-varying use of additional antipsychotic medications in each 3-month period of follow-up time, and baseline variables such as age, sex, time since psychotic disorder diagnosis, decile of the time-conditional propensity score, prior year hospital admissions, being accused of a crime, diagnosis of personality disorder, substance use disorder, and mood/anxiety disorder.

SG-LAIA, second-generation long-acting injectable antipsychotic.

### Sensitivity Analysis

Results in the cohort of SG-LAIA new users who had a diagnosis of schizophrenia were similar, with a few notable exceptions (Supplementary Figures S1, S2). There was no observed reduction in all-cause mortality (adjusted hazard ratio 0.79 and 95% hazard ratio 0.55–1.12), and there was a reduced risk of psychiatric hospitalization during monotherapy (adjusted hazard ratio 0.81 and 95% confidence interval 0.72–0.92). *Post hoc* sensitivity analysis where a prior antipsychotic was included in the time-conditional propensity score improved the baseline balance in the number of subjects previously treated with quetiapine and aripiprazole, with a minimal change in hazard ratios (Supplementary Tables S6, S7).

## Discussion

We used a prevalent new-user cohort design to evaluate the effectiveness of switching to an SG-LAIA compared with continuing an oral antipsychotic treatment regimen. In the overall cohort, we found the risk of treatment failure, incarceration, and treatment discontinuation was similar in SG-LAIA and oral antipsychotic users; the risk of psychiatric hospitalization was increased in SG-LAIA users, but the risk of all-cause mortality was decreased. Subsets of this population-based cohort benefitted from the SG-LAIA prescription, notably those receiving antipsychotic monotherapy and those who had no prior year of antipsychotic use, with the risk of treatment failure reduced by 17 and 43%, respectively. In contrast, prevalent antipsychotic users who switched to SG-LAIAs were found to have an increased risk of treatment failure, psychiatric hospitalization, and treatment discontinuation. Previous research has established that LAIAs have a greater benefit when used early in the course of the disease, but there is also evidence of effectiveness in prevalent antipsychotic users (Alphs et al., 2016; Tiihonen et al., 2017). Despite matching with the propensity score and a good balance of measured baseline variables including the duration of illness, prior antipsychotic exposure, and hospitalizations, we cannot rule out that this observation may be confounded. Patients switching to SG-LAIAs may not be comparable to those who were stabilized on a prior antipsychotic regimen. Crude hazard ratios shifted after adjusting for additional covariates, so the prevalent new-user design and propensity score matching were not sufficient to control confounding.

Other observational studies have found that the SG-LAIA use reduces the risk of treatment failure, treatment discontinuation, hospitalization, and mortality compared with oral antipsychotics (Tiihonen et al., 2011, 2017; Stip and Lachaine, 2018; Taipale et al., 2018; Song et al., 2019). Tiihonen et al. observed adjusted hazard ratios for the risk of treatment failure during monotherapy with paliperidone-LAI and risperidone-LAI of 0.80 and 0.72, respectively, compared with oral olanzapine monotherapy in a Swedish population-based cohort (Tiihonen et al., 2017). Taipale et al. also demonstrated an increased risk of mortality with oral antipsychotics and FG-LAIAs compared to SG-LAIAs in the same Swedish cohort (adjusted hazard ratio 1.51 for oral SGAs, 1.83 for oral FGAs, and 1.37 for FG-LAIAs) (Taipale et al., 2018). Despite the demonstrated benefits of LAIA treatment, event rates were considerable. Our study estimated crude incidence rates of approximately 60 treatment failure events, 25 psychiatric hospitalizations, and 38 treatment discontinuation events per 100 person years of SG-LAIA exposure. Similar or higher rates were observed in the Swedish cohort for treatment failure (IR 9.3 and 6.4 per 10 person years for paliperidone- and risperidone-LAI, respectively) and psychiatric hospitalization (IR 5.1 and 3.8 per 10 person years for paliperidone and risperidone LAI, respectively) (Tiihonen et al., 2017).

We observed a non-significant trend toward reduction in the risk of incarceration in the overall cohort and a remarkable 68% reduction in the risk of incarceration in incident new users of SG-LAIA. This finding is in line with previous research, including a pragmatic randomized trial that showed paliperidone-LAI reduced time to incarceration (Alphs et al., 2015) and a cohort study showing a 70% reduction in the risk of violent crimes during LAIA treatment (Fazel et al., 2014).

While observational designs of SG-LAIA effectiveness can be subject to unmeasured confounding, the direction of bias in this study is most likely in favor of an active comparator for a few reasons. First, LAIA users have been shown to have more severe diseases than patients who were not prescribed LAIAs (Kishimoto et al., 2018). Second, in Manitoba, SG-LAIA agents are reserved as second-line agents, for patients with evidence of non-adherence, treatment failure, or intolerance to another antipsychotic. Third, the increased frequency of contact between SG-LAIA users and healthcare providers introduces detection bias, as the need for hospitalization or treatment escalation is detected earlier in patients who are monitored more frequently. Thus, we are more confident in our results that show significant reductions in the risk of outcomes associated with SG-LAIA use than we are in those that show an increased risk.

This study has numerous strengths. By using a prevalent new-user design, we were able to increase our sample size by almost 1,000 patients. The data used from the Manitoba Population Research Data Repository have undergone a rigorous quality assessment, and we used established definitions to identify comorbidities and outcomes (Chartier et al., 2018; Smith et al., 2018). We had a 20-year study period with over 3,100 person years of observation time. We included incarceration as a reason for treatment failure and adjusted for having been accused of a crime. Finally, we have previously validated SG-LAIA exposure in prescription claim data (Janzen et al., 2022).

This study reinforces the evidence from previous work, suggesting LAIAs are superior to oral antipsychotics at the early stages of the disease and during monotherapy. We encourage clinicians to offer SG-LAIA treatment to all patients initiating antipsychotic therapy. However, it remains unclear whether there is a benefit to switching stable patients from oral antipsychotics to SG-LAIAs.

## Conclusion

In this population-based cohort study, the SG-LAIA use was not associated with a reduced risk of treatment failure compared with other antipsychotics but did reduce mortality. Monotherapy with SG-LAIAs and the incident use of SG-LAIAs were associated with a reduced risk of treatment failure.

## Data Availability

The data analyzed in this study are subject to the following licenses/restrictions. Data used in this article were derived from administrative health and social data as a secondary use. The data were provided under specific data sharing agreements only for approved use at the Manitoba Centre for Health Policy (MCHP). The original source data are not owned by the researchers or MCHP and as such cannot be provided to a public repository. The original data source and approval for use have been noted in the acknowledgments of the article. If necessary, source data specific to this article or project may be reviewed at MCHP with the consent of the original data providers, along with the required privacy and ethical review bodies. Requests to access these datasets should be directed to the Manitoba Centre for Health Policy, https://umanitoba.ca/manitoba-centre-for-health-policy/data-repository.
