# Supplementary material for: Second-Generation Long-Acting Injectable Antipsychotics and the Risk of Treatment Failure in a Population-Based Cohort
Source: Front Pharmacol. 2022 May 19;13:879224. doi: 10.3389/fphar.2022.879224 (PMC9160742; doi:10.3389/fphar.2022.879224)

Supplement to: Second-generation long-acting injectable antipsychotics and risk of treatment failure in a population-based cohort

Supplementary Table 1. Exposure and outcome definitions.

Variable	Definition
Medications	
Alcohol use disorder drug	ATC code beginning with N07BB appearing at least once in prescription claims
Anticholinergic	ATC code beginning with N04AA, N04AB, N04AC appearing at least once in prescription claims
Anticonvulsant	ATC code beginning with N03A, excluding N03AE01 (clonazepam) appearing at least once in prescription claims
Antidepressant	ATC code beginning with N06A appearing at least once in prescription claims
Antidiabetic drug	ATC code beginning with A10A, A10B, A10X appearing at least once in prescription claims
Antihyperlipidemic drug	ATC code beginning with C10A, C10B appearing at least once in prescription claims
Antipsychotic	ATC code beginning with N05A, excluding ATC N05AN01 (lithium) appearing at least once in prescription claims
Anxiolytic	ATC code beginning with N05BA; ATC codes N03AE01, N05BE01 appearing at least once in prescription claims
Dementia drug	ATC code beginning with N06DA, N06DX appearing at least once in prescription claims
Long-acting injectable antipsychotic	ATC code beginning with N05A, excluding ATC N05AN01 (lithium) and route intramuscular or subcutaneous, excluding non-LAIA injectables (DINs 00017574, 00808652, 02366010, 00743518, 01929984, 00497509, 01927698, 00028002, 01927795, 02162946, 02230405, 00439819, 02169991, 02247099, 01933272, 00627615, 02382016, 02167832, 02232966, 02406411, 02416522, 02232449) appearing at least once in prescription claims
Mood stabilizer	ATC codes N03AF01, N03AF02, N03AG01, N03AX09, N05AN01 appearing at least once in prescription claims
Opioid	ATC code beginning with N02A; ATC codes N07BC02, R05DA01, R05DA03-06, R05DA12, R05DA20, R05FA01, R05FA02 appearing at least once in prescription claims
Opioid agonist therapy	ATC code beginning with N07BC appearing at least once in prescription claims
Sedative-hypnotic	ATC code beginning with N05C, R06AA; ATC codes N05BB01, R06AD02, N06AX02, N05CH01 appearing at least once in prescription claims
Smoking cessation aid	ATC codes N07BA01, N07BA03; DIN 02238441 appearing at least once in prescription claims
Diagnoses	
Attention deficit/hyperactivity disorder	ICD-9-CM code 314 or ICD-10-CA code F90 appearing at least once in hospital claims; or<br>ICD-9-CM code 314 appearing at least once in medical claims; or<br>ATC code N06BA appearing at least twice in prescription claims without ICD-9-CM 312, 313, 347 or ICD-10-CA F63, F91, F92, F93, F94, G47.4 in hospital or medical claims; or<br>ATC code N06BA appearing at least once in prescription claims and ICD-9-CM code 314 or ICD-10-CA code F90 appearing at least once in hospital or medical claims in the previous 3 years
Autism spectrum disorder	ICD-9-CM code 299.0, 299.1, 299., 299.9 or ICD-10-CA code F84.0, F84.1, F84.2, F84.3, F84.4, F84.5, F84.8, F84.9 appearing at least once in hospital claims; or<br>ICD-9-CM code 299 appearing at least once in medical claims

Dementia	ICD-9-CM code 290, 291.1, 292.2, 292.82, 294, 331, 797 or ICD-10-CA code F00, F01, F02, F03, F04, F05.1, F06.5, F06.6, F06.8, F06.9, F09, F10.7, F11.7, F12.7, F14.7, F15.7, F18.7, F19.7, G30, G31.1, G31.9, G32.8, G91, G93.7, G94, R54 appearing at least once in hospital claims; or<br>ICD-9-CM code 290, 294, 331, 797 appearing at least once in medical claims
Intellectual disability/ developmental disorder	ICD-9-CM code 299, 317, 318, 319, 758.0, 758.1, 758.2, 758.3, 759.81, 759.82, 759.83, 759.84, 759.85, 759.86, 759.87, 759.88, 759.89, 760.71 or ICD-10-CA code F70.0, F70.1, F70.8, F70.9, F71.0, F71.1, F71.8, F71.9, F72.0, F72.1, F72.8, F72.9, F73.0, F73.1, F73.8, F73.9, F78.0, F78.1, F78.8, F78.9, F79.0, F79.1, F79.8, F79.9, F84.0, F84.1, F84.3, F84.4, F84.5, F84.8, F84.9, Q86.0, Q86.1, Q86.2, Q86.8, Q87.0, Q87.1, Q87.2, Q87.3, Q87.5, Q87.8, Q89.8, Q90.0, Q90.1, Q90.9, Q91.0, Q91.1, Q91.2, Q91.3, Q91.4, Q91.5, Q91.6, Q91.7, Q93.0, Q93.1, Q93.2, Q93.3, Q93.4, Q93.5, Q93.6, Q93.7, Q93.8, Q93.9, Q99.2 appearing at least once in hospital claims; or<br>ICD-9-CM code 317, 318, 319, 299 appearing at least once in medical claims
Mood or anxiety disorder	ICD-9-CM code 296, 300.0, 300.2, 300.3, 300.4, 300.7 309, 311 or ICD-10-CA code F31, F32, F33, F34.1, F38.0, F38.1, F40, F41.0, F41.1, F41.2, F41.3, F41.8, F41.9, F42, F43.1, F43.2, F43.8, F45.2, F53.0, F93.0 appearing at least once in hospital claims; or<br>ICD-9-CM code 300 or ICD-10-CA code F41, F44, F45.0, F45.1, F68.0 or F99 appearing at least once in hospital claims and ATC code N05AN01, N05BA or N06A appearing at least once in prescription claims; or<br>ICD-9-CM code 296, 311 appearing at least once in medical claims; or<br>ICD-9-CM code 300 appearing at least once in medical claims and ATC code N05AN01, N05BA or N06A appearing at least once in prescription claims; or<br>ICD-9-CM code 300, 309 appearing at least 3 times in medical claims
Personality disorder	ICD-9-CM code 301 or ICD-10-CA code F34.0, F60, F61, F62, F68.1, F68.8, F69 appearing at least once in hospital claims; or<br>ICD-9-CM code 301 appearing at least once in medical claims
Psychotic disorder	ICD-9-CM code 295, 297, 298 or ICD-10-CA code F11.5, F12.5, F13.5, F14.5, F15.5, F16.5, F18.5, F19.5, F20, F22, F23, F24, F25, F28 or F29 appearing at least once in hospital claims; or<br>ICD-9-CM code 295, 297, 298 appearing at least once in medical claims
Schizophrenia	ICD-9-CM code 295 or ICD-10-CA code F20 appearing at least once in hospital claims; or<br>ICD-9-CM code 295 appearing at least once in medical claims
Substance use disorder	ICD-9-CM code 291, 292, 303, 304, 305 or ICD-10-CA code F10, F11, F12, F13, F14, F15, F16, F17, F18, F19, F55, Z50.2, Z50.3 appearing at least once in hospital claims; or<br>ICD-9-CM code 291, 292, 303, 304, 305 appearing at least once in medical claims
Suicide attempt	ICD-9-CM code E95.0-95.9 or ICD-10-CA code X60-84 appearing at least once in hospital claims; or<br>ICD-9-CM code 965, 967, 969, 977.9, 986, E850-854, E858, E862, E868 or ICD-10-CA code T39, T40, T42.3, T42.4, T42.7, T43, T50.9, T58, X40-42, X44, X46, X47, Y10-12, Y16, Y17 appearing at least once in hospital claims and tariff code 8444, 8446, 8472, 8475, 8476, 8503, 8504, 8553, 8554, 8581, 8584, 8588, 8596, 8580, 8587 or 8589 appearing in hospital claims during admission or in medical claims within 30 days of hospital separation
Other baseline variables	

Accused of a crime	Identified in the Prosecutions Information and Scheduling Management database as having been accused of a crime, excluding offenses under the Highway Traffic Act
Income quintile	Area-level income based on postal code of residence
Victim of a crime	Identified in the Prosecutions Information and Scheduling Management database as having been victim of a crime, excluding offenses under the Highway Traffic Act
Outcomes	
Completed suicide	Primary cause of death self-inflicted injury or poisoning, or poisoning of undetermined intent (ICD-9-CM code E950-952, E953-958, E959 or ICD-10-CA code X40-42, X46, X47, X70-X84, Y10-12, Y16-17, Y87.0)
Incarceration	Identified in the Corrections Offender Management System database as having been taken into custody, excluding probation and intoxicated persons detention
Psychiatric hospitalization	Hospitalization for a mood or anxiety disorder, substance use disorder, psychotic disorder, schizophrenia or attempted suicide (ICD-9-CM codes 296, 311, 309, 300, 291, 292, 303, 304,3 05, 295, 301, E95.0-95.9 or ICD-10-CA F30, F31, F32, F33, F34, F38, F40, F41.0, F41.1, F41.2, F41.3, F41.8, F41.9, F42, F43, F53.0, F10-19, F55, Z50.2, Z50.3, F20, F21, F60-62, F69, X60-84; or ICD-9-CM code 965, 967, 969, 977.9, 986, E850-854, E858, E862, E868 or ICD-10-CA code T39, T40, T42.3, T42.4, T42.7, T43, T50.9, T58, X40-42, X44, X46, X47, Y10-12, Y16, Y17 and tariff code 8444, 8446, 8472, 8475, 8476, 8503, 8504, 8553, 8554, 8581, 8584, 8588, 8596, 8580, 8587 or 8589 appearing in hospital claims during admission or in medical claims within 30 days of hospital separation)
Treatment discontinuation	Gap of >90 days between consecutive dispensations in prescription claims data

ATC=anatomical therapeutic chemical; DIN=drug identification number; ICD-9-CM=International Classification of Diseases, Ninth Revision, Clinical Modification; ICD-10-CA=International Classification of Diseases, Tenth Revision, with Canadian Enhancements; LAIA=long-acting injectable antipsychotic

Supplementary Table 2. Antipsychotics used by cohort members prior to and upon cohort entry.

	Before matching					After matching				
	SG-LAIA New Users, n=1,681		Antipsychotic Users, n=1,681*		Standard-ized	SG-LAIA New Users, n=1,182		Antipsychotic Users, n=1,182		Standard-ized
Characteristics	n or mean	% or SD	n or mean	% or SD	Difference	n or mean	% or SD	n or mean	% or SD	Difference
Prevalent antipsychotic used prior to cohort entry†										
SGA	1258	74.8%	1168	69.5%	0.16	881	74.5%	862	72.9%	0.06
Aripiprazole	117	7.0%	29	1.7%	0.28	80	6.8%	30	2.5%	0.22
Brexpiprazole	S	S	S	S	0.04	S	S	0	0.0%	0.04
Clozapine	83	4.9%	66	3.9%	0.05	52	4.4%	36	3.0%	0.08
Lurasidone	S	S	S	S	0.03	S	S	S	S	0.02
Olanzapine	360	21.4%	309	18.4%	0.08	252	21.3%	239	20.2%	0.03
Paliperidone	6	0.4%	S	S	0.09	S	S	S	S	0.04
Quetiapine	274	16.3%	378	22.5%	0.17	190	16.1%	277	23.4%	0.21
Risperidone	402	23.9%	384	22.8%	0.03	290	24.5%	277	23.4%	0.03
Ziprasidone	11	0.7%	S	S	0.12	10	0.8%	0	0.0%	0.14
FGA	221	13.1%	311	18.5%	0.16	114	9.6%	133	11.3%	0.06
Chlorpromazine	7	0.4%	42	2.5%	0.19	S	S	10	0.8%	0.07
Flupentixol	10	0.6%	15	0.9%	0.04	S	S	6	0.5%	0.04
Flupentixol LAI	35	2.1%	52	3.1%	0.07	18	1.5%	22	1.9%	0.03
Fluphenazine	S	S	7	0.4%	0.08	0	0.0%	S	S	0.09
Fluphenazine LAI	38	2.3%	37	2.2%	0.00	22	1.9%	11	0.9%	0.09
Haloperidol	38	2.3%	27	1.6%	0.05	25	2.1%	15	1.3%	0.07
Haloperidol LAI	38	2.3%	26	1.5%	0.06	12	1.0%	13	1.1%	0.01

Loxapine	8	0.5%	8	0.5%	0.00	S	S	8	0.7%	0.05
Methotrimeprazine	10	0.6%	48	2.9%	0.19	6	0.5%	17	1.4%	0.10
Perphenazine	S	S	S	S	0.02	S	S	S	S	0.04
Pimozide	6	0.4%	S	S	0.01	6	0.5%	S	S	0.06
Pipotiazine palmitate LAI	S	S	S	S	0.04	S	S	0	0.0%	0.08
Thiothixene	S	S	0	0.0%	0.04	0	0.0%	0	0.0%	0.00
Trifluoperazine	S	S	20	1.2%	0.12	S	S	7	0.6%	0.07
Zuclopenthixol	6	0.4%	S	S	0.07	S	S	0	0.0%	0.06
Zuclopenthixol LAI	15	0.9%	20	1.2%	0.03	6	0.5%	15	1.3%	0.09
None	202	12.0%	202	12.0%	0.00	187	15.8%	187	15.8%	0.00
Antipsychotic dispensed on t_0										
SG-LAIA										
Aripiprazole LAI	-	-	-	-	-	140	11.8%	-	-	-
Paliperidone LAI‡	-	-	-	-	-	455	38.5%	-	-	-
Risperidone LAI	-	-	-	-	-	587	49.7%	-	-	-
SGA	-	-	-	-	-	-	-	1027	86.9%	-
Aripiprazole	-	-	-	-	-	-	-	44	3.7%	-
Brexpiprazole	-	-	-	-	-	-	-	S	S	-
Clozapine	-	-	-	-	-	-	-	36	3.0%	-
Lurasidone	-	-	-	-	-	-	-	S	S	-
Olanzapine	-	-	-	-	-	-	-	290	24.5%	-
Paliperidone	-	-	-	-	-	-	-	S	S	-
Quetiapine	-	-	-	-	-	-	-	309	26.1%	-

Risperidone	-	-	-	-	-	-	-	344	29.1%	-
Ziprasidone	-	-	-	-	-	-	-	S	S	-
FGA	-	-	-	-	-	-	-	155	13.1%	-
Chlorpromazine	-	-	-	-	-	-	-	11	0.9%	-
Flupentixol	-	-	-	-	-	-	-	6	0.5%	-
Flupentixol LAI	-	-	-	-	-	-	-	24	2.0%	-
Fluphenazine	-	-	-	-	-	-	-	S	S	-
Fluphenazine LAI	-	-	-	-	-	-	-	13	1.1%	-
Haloperidol	-	-	-	-	-	-	-	25	2.1%	-
Haloperidol LAI	-	-	-	-	-	-	-	16	1.4%	-
Loxapine	-	-	-	-	-	-	-	9	0.8%	-
Methotrimeprazine	-	-	-	-	-	-	-	19	1.6%	-
Perphenazine	-	-	-	-	-	-	-	S	S	-
Pimozide	-	-	-	-	-	-	-	S	S	-
Trifluoperazine	-	-	-	-	-	-	-	7	0.6%	-
Zuclopenthixol	-	-	-	-	-	-	-	0	0.0%	-
Zuclopenthixol LAI	-	-	-	-	-	-	-	16	1.4%	-
Total FG-LAIA	-	-	-	-	-	-	-	69	5.8%	

*Cohort before matching consisted of SG-LAIA new users and antipsychotic users in the exposure set of an SG-LAIA new user. Exposure sets were based on calendar time, prior duration of continuous antipsychotic use, prior year use of clozapine, prior use of FG-LAIA and prior year number of unique antipsychotic medications dispensed. Characteristics of a random sample of one antipsychotic user per exposure set are reported. SG-LAIA users were matched 1:1 with an antipsychotic user on time-conditional propensity score.

†If multiple prior antipsychotics were used, the antipsychotic with longest duration of continuous use was selected

‡Includes once-monthly and 3-monthly formulations of paliperidone

FGA=first-generation antipsychotic; FG-LAIA=first-generation long-acting injectable antipsychotic; LAI=long-acting injectable; S=suppressed due to count <6; SD=standard deviation; SGA=second-generation antipsychotic; SG-LAIA=second-generation long-acting injectable antipsychotic; t_0=cohort entry date

Supplementary Table 3. Baseline characteristics of incident and prevalent new user cohort members before and after matching.

Characteristics	Before Matching										After Matching									
	SG-LAI New Users, n=1,681				Antipsychotic Users, n=1,681*				Standardized Difference		SG-LAI New Users, n=1,182				Antipsychotic Users, n=1,182				Standardized Difference	
	Incident New Users, n=202		Prevalent New Users, n=1,479		Incident Users, n=202*		Prevalent Users, n=1,479*		Incident Users	Prev-alent Users	Incident New Users, n=187		Prevalent New Users, n=995		Incident Users, n=187		Prevalent Users, n=995		Incident Users	Prev-alent Users
	n or mean	% or SD	n or mean	% or SD	n or mean	% or SD	n or mean	% or SD			n or mean	% or SD	n or mean	% or SD	n or mean	% or SD	n or mean	% or SD		
Females	79	39.1%	525	35.5%	86	42.6%	643	43.5%	0.07	0.16	74	39.6%	361	36.3%	86	46.0%	380	38.2%	0.13	0.04
Age (years)	37	17.1	36	16.2	50	26.1	50	20.2	0.58	0.75	37	17.0	37	17.2	35	17.2	37	17.4	0.11	0.02
Age group (years)																				
<18	S	S	44	3.0%	16	7.9%	54	3.7%	0.22	0.04	6	3.2%	32	3.2%	16	8.6%	95	9.5%	0.23	0.26
18-30	96	47.5%	661	44.7%	49	24.3%	265	17.9%	0.50	0.60	90	48.1%	434	43.6%	81	43.3%	343	34.5%	0.10	0.19
31-40	29	14.4%	293	19.8%	31	15.3%	190	12.8%	0.03	0.19	26	13.9%	190	19.1%	39	20.9%	198	19.9%	0.18	0.02
41-50	22	10.9%	196	13.3%	16	7.9%	263	17.8%	0.10	0.13	20	10.7%	128	12.9%	14	7.5%	164	16.5%	0.11	0.10
51-60	27	13.4%	150	10.1%	16	7.9%	284	19.2%	0.18	0.26	25	13.4%	104	10.5%	21	11.2%	99	9.9%	0.07	0.02
61-70	12	5.9%	78	5.3%	12	5.9%	186	12.6%	0.00	0.26	10	5.3%	55	5.5%	7	3.7%	44	4.4%	0.08	0.05
71-80	6	3.0%	30	2.0%	22	10.9%	91	6.2%	0.32	0.21	S	S	28	2.8%	S	S	19	1.9%	0.07	0.06
>80	S	S	27	1.8%	40	19.8%	146	9.9%	0.60	0.35	S	S	24	2.4%	S	S	33	3.3%	0.03	0.05
Income quintile																				
1 (lowest)	76	37.6%	546	36.9%	72	35.6%	525	35.5%	0.04	0.03	73	39.0%	376	37.8%	79	42.2%	378	38.0%	0.07	0.00
2	35	17.3%	311	21.0%	35	17.3%	334	22.6%	0.00	0.04	34	18.2%	218	21.9%	39	20.9%	202	20.3%	0.07	0.04
3	25	12.4%	189	12.8%	23	11.4%	209	14.1%	0.03	0.04	24	12.8%	128	12.9%	16	8.6%	127	12.8%	0.14	0.00
4	26	12.9%	166	11.2%	30	14.9%	139	9.4%	0.06	0.06	25	13.4%	115	11.6%	26	13.9%	119	12.0%	0.02	0.01
5 (highest)	14	6.9%	113	7.6%	22	10.9%	141	9.5%	0.14	0.07	13	7.0%	80	8.0%	12	6.4%	97	9.7%	0.02	0.06
Missing	26	12.9%	154	10.4%	20	9.9%	131	8.9%	0.09	0.05	18	9.6%	78	7.8%	15	8.0%	72	7.2%	0.06	0.02
Year of cohort entry																				
2005/2006	6	3.0%	39	2.6%	7	3.5%	42	2.8%	0.03	0.01	S	S	36	3.6%	S	S	35	3.5%	0.00	0.01
2007/2008	8	4.0%	73	4.9%	7	3.5%	67	4.5%	0.03	0.02	S	S	52	5.2%	S	S	53	5.3%	0.00	0.00
2009/2010	13	6.4%	92	6.2%	14	6.9%	97	6.6%	0.02	0.01	11	5.9%	63	6.3%	13	7.0%	61	6.1%	0.04	0.01
2011/2012	22	10.9%	171	11.6%	21	10.4%	162	11.0%	0.02	0.00	22	11.8%	102	10.3%	21	11.2%	108	10.9%	0.02	0.02
2013/2014	25	12.4%	181	12.2%	28	13.9%	167	11.3%	0.04	0.01	23	12.3%	106	10.7%	22	11.8%	100	10.1%	0.02	0.02

2015/2016	34	16.8%	329	22.2%	31	15.3%	362	24.5%	0.04	0.00	30	16.0%	216	21.7%	32	17.1%	211	21.2%	0.03	0.01
2017/2018	53	26.2%	360	24.3%	55	27.2%	359	24.3%	0.02	0.00	51	27.3%	233	23.4%	49	26.2%	249	25.0%	0.02	0.04
2019/2020	41	20.3%	254	17.2%	39	19.3%	255	17.2%	0.02	0.00	37	19.8%	187	18.8%	37	19.8%	178	17.9%	0.00	0.02
Time since psychotic disorder diagnosis (years)	5.7	6.3	8.4	6.7	4.0	5.9	10.9	7.7	0.28	0.35	5.4	6.1	7.6	6.7	5.1	6.2	7.6	6.7	0.05	0.01
<1	74	36.6%	163	11.0%	92	45.5%	145	9.8%	0.18	0.04	74	39.6%	155	15.6%	72	38.5%	156	15.7%	0.02	0.00
1-4.9	41	20.3%	458	31.0%	59	29.2%	342	23.1%	0.21	0.18	38	20.3%	337	33.9%	45	24.1%	323	32.5%	0.09	0.03
5-10	32	15.8%	309	20.9%	21	10.4%	208	14.1%	0.16	0.18	27	14.4%	170	17.1%	30	16.0%	179	18.0%	0.04	0.02
>10	55	27.2%	549	37.1%	30	14.9%	784	53.0%	0.31	0.32	48	25.7%	333	33.5%	72	38.5%	337	33.9%	0.10	0.01
Prior FG-LAIA use	21	10.4%	368	24.9%	21	10.4%	368	24.9%	0.00	0.00	16	8.6%	136	13.7%	16	8.6%	136	13.7%	0.00	0.00
Prior year antipsychotic medications																				
0-1	202	100.0%	597	40.4%	202	100.0%	597	40.4%	0.00	0.00	187	100.0%	514	51.7%	187	100.0%	514	51.7%	0.00	0.00
>1	0	0.0%	749	50.6%	0	0.0%	749	50.6%	0.00	0.00	0	0.0%	407	40.9%	0	0.0%	407	40.9%	0.00	0.00
Clozapine	0	0.0%	133	9.0%	0	0.0%	133	9.0%	0.00	0.00	0	0.0%	74	7.4%	0	0.0%	74	7.4%	0.00	0.00
Prior year number of medication classes dispensed																				
0-1	118	58.4%	336	22.7%	43	21.3%	200	13.5%	0.82	0.24	105	56.1%	233	23.4%	109	58.3%	210	21.1%	0.04	0.06
2-5	56	27.7%	548	37.1%	73	36.1%	460	31.1%	0.18	0.13	55	29.4%	377	37.9%	53	28.3%	377	37.9%	0.02	0.00
>5	28	13.9%	595	40.2%	86	42.6%	819	55.4%	0.67	0.31	27	14.4%	385	38.7%	25	13.4%	408	41.0%	0.03	0.05
Prior year medication use																				
Mood stabilizer	7	3.5%	273	18.5%	16	7.9%	293	19.8%	0.19	0.03	7	3.7%	181	18.2%	S	S	166	16.7%	0.13	0.04
Antidepressant	28	13.9%	567	38.3%	80	39.6%	715	48.3%	0.06	0.20	28	15.0%	386	38.8%	21	11.2%	431	43.3%	0.11	0.09
Anxiolytic	26	12.9%	586	39.6%	54	26.7%	647	43.7%	0.35	0.08	25	13.4%	378	38.0%	25	13.4%	385	38.7%	0.00	0.01
Sedative-hypnotic	18	8.9%	302	20.4%	38	18.8%	374	25.3%	0.29	0.12	18	9.6%	209	21.0%	17	9.1%	208	20.9%	0.02	0.00
Anticonvulsant	13	6.4%	88	5.9%	15	7.4%	157	10.6%	0.20	0.17	6	3.2%	64	6.4%	S	S	59	5.9%	0.10	0.02
Psychostimulant	S	S	49	3.3%	S	S	14	0.9%	0.00	0.16	S	S	31	3.1%	S	S	36	3.6%	0.05	0.03
Anticholinergic	13	6.4%	384	26.0%	12	5.9%	320	21.6%	0.21	0.10	12	6.4%	221	22.2%	10	5.3%	214	21.5%	0.10	0.02
Opioid	23	11.4%	301	20.4%	47	23.3%	324	21.9%	0.32	0.04	23	12.3%	197	19.8%	18	9.6%	204	20.5%	0.09	0.02
Opoid agonist therapy	S	S	12	0.8%	S	S	S	S	0.00	0.07	S	S	7	0.7%	S	S	11	1.1%	0.10	0.04
Smoking cesation aid	S	S	61	4.1%	S	S	66	4.5%	0.04	0.02	S	S	41	4.1%	S	S	40	4.0%	0.10	0.01

Alcohol use disorder drug	0	0.0%	S	S	S	S	S	S	0.10	0.08	0	0.0%	S	S	0	0.0%	S	S	0.00	0.03
Dementia drug	S	S	S	S	16	7.9%	49	3.3%	0.38	0.22	S	S	S	S	0	0.0%	S	S	0.10	0.03
Antidiabetic drug	6	S	159	10.8%	16	7.9%	262	17.7%	0.22	0.20	6	3.2%	97	9.7%	S	S	103	10.4%	0.03	0.02
Antihyperlipidemic drug	S	S	125	8.5%	36	17.8%	263	17.8%	0.53	0.28	S	S	81	8.1%	S	S	89	8.9%	0.03	0.03
Comorbidities																				
Mood or anxiety disorder	121	59.9%	1162	78.6%	146	72.3%	1225	82.8%	0.29	0.11	116	62.0%	790	79.4%	121	64.7%	795	79.9%	0.06	0.01
Personality disorder	45	22.3%	440	29.7%	47	23.3%	425	28.7%	0.02	0.02	41	21.9%	267	26.8%	43	23.0%	265	26.6%	0.03	0.00
Substance use disorder	91	45.0%	876	59.2%	82	40.6%	644	43.5%	0.09	0.32	88	47.1%	550	55.3%	89	47.6%	543	54.6%	0.01	0.01
Dementia	20	9.9%	181	12.2%	63	31.2%	376	25.4%	0.55	0.34	20	10.7%	126	12.7%	14	7.5%	145	14.6%	0.11	0.06
Autism spectrum disorder	S	S	36	2.4%	S	S	39	2.6%	0.08	0.01	S	S	26	2.6%	S	S	27	2.7%	0.09	0.01
Intellectual disability/ developmental disorder	14	6.9%	185	12.5%	10	5.0%	128	8.7%	0.08	0.13	13	7.0%	104	10.5%	8	4.3%	108	10.9%	0.12	0.01
ADHD	S	S	228	15.4%	15	7.4%	105	7.1%	0.04	0.27	12	6.4%	137	13.8%	13	7.0%	158	15.9%	0.02	0.06
Suicide attempt	14	6.9%	170	11.5%	S	S	136	9.2%	0.13	0.08	14	7.5%	93	9.3%	20	10.7%	100	10.1%	0.11	0.02
Prior year hospitalizations																				
0	42	20.8%	432	29.2%	105	52.0%	959	64.8%	0.69	0.76	42	22.5%	351	35.3%	50	26.7%	341	34.3%	0.10	0.02
1-2	152	75.2%	821	55.5%	S	S	458	31.0%	0.64	0.51	137	73.3%	550	55.3%	130	69.5%	560	56.3%	0.08	0.02
>2	8	4.0%	226	15.3%	S	S	62	4.2%	0.08	0.38	8	4.3%	94	9.4%	7	3.7%	94	9.4%	0.03	0.00
Prior year physician visits																				
0-2	20	9.9%	41	2.8%	16	7.9%	93	6.3%	0.07	0.17	20	10.7%	34	3.4%	30	16.0%	34	3.4%	0.16	0.00
3-5	24	11.9%	103	7.0%	17	8.4%	137	9.3%	0.11	0.08	24	12.8%	73	7.3%	20	10.7%	69	6.9%	0.07	0.02
>5	158	78.2%	1335	90.3%	169	83.7%	1249	84.4%	0.14	0.18	143	76.5%	888	89.2%	137	73.3%	892	89.6%	0.07	0.01
Incidents where accused of a crime																				
0	137	67.8%	1019	68.9%	154	76.2%	1321	89.3%	0.31	0.52	129	69.0%	722	72.6%	132	70.6%	745	74.9%	0.03	0.05
1-2	28	13.9%	204	13.8%	23	11.4%	90	6.1%	0.07	0.26	25	13.4%	128	12.9%	29	15.5%	122	12.3%	0.06	0.02
>2	37	18.3%	256	17.3%	15	7.4%	68	4.6%	0.33	0.42	33	17.6%	145	14.6%	26	13.9%	128	12.9%	0.10	0.05
Incidents where victim of a crime																				
0	193	95.5%	1392	94.1%	S	S	1432	96.8%	0.14	0.13	179	95.7%	950	95.5%	179	95.7%	945	95.0%	0.00	0.02
>0	9	4.5%	87	5.9%	S	S	47	3.2%	0.14	0.13	8	4.3%	45	4.5%	8	4.3%	50	5.0%	0.00	0.02

*Cohort before matching consisted of SG-LAIA new users and antipsychotic users in the exposure set of an SG-LAIA new user. Exposure sets were based on calendar time, prior duration of continuous antipsychotic use, prior year use of clozapine, prior use of FG-LAIA and prior year number of unique antipsychotic medications dispensed. Characteristics of a random sample of one antipsychotic user per exposure set are reported. SG-LAIA users were matched 1:1 with an antipsychotic user on time-conditional propensity score.

Supplementary Table 4. Baseline characteristics of matched cohort members included in monotherapy subgroup.

	After matching				
	SG-LAIA New Users, n=519		Antipsychotic Users, n=519		
Characteristics	n or mean	% or SD	n or mean	% or SD	Standardized Difference
Females	187	36.0%	214	41.2%	0.11
Age (years)	36.4	16.7	36.2	17.6	0.01
Age group (years)					
<18	45	8.7%	12	2.3%	0.28
18-30	206	39.7%	240	46.2%	0.13
31-40	104	20.0%	96	18.5%	0.04
41-50	67	12.9%	58	11.2%	0.05
51-60	39	7.5%	60	11.6%	0.14
61-70	26	5.0%	32	6.2%	0.05
71-80	13	2.5%	11	2.1%	0.03
>80	19	3.7%	10	1.9%	0.11
Income quintile					
1 (lowest)	203	39.1%	198	38.2%	0.11
2	100	19.3%	85	16.4%	0.08
3	50	9.6%	76	14.6%	0.15
4	73	14.1%	74	14.3%	0.01
5 (highest)	53	10.2%	44	8.5%	0.06
Missing	40	7.7%	42	8.1%	0.01
Year of cohort entry					

2005/2006	19	3.7%	19	3.7%	0.00
2007/2008	25	4.8%	25	4.8%	0.00
2009/2010	37	7.1%	40	7.7%	0.02
2011/2012	54	10.4%	54	10.4%	0.00
2013/2014	52	10.0%	49	9.4%	0.02
2015/2016	103	19.8%	103	19.8%	0.00
2017/2018	137	26.4%	133	25.6%	0.02
2019/2020	92	17.7%	96	18.5%	0.02
Time since psychotic disorder diagnosis (years)	6.9	6.6	6.4	6.4	0.07
<1	132	25.4%	106	20.4%	0.12
1-4.9	152	29.3%	167	32.2%	0.06
5-10	92	17.7%	83	16.0%	0.05
>10	142	27.4%	163	31.4%	0.09
Prior FG-LAIA use	59	11.4%	59	11.4%	0.00
Prior year antipsychotic medications					
0-1	381	73.4%	381	73.4%	0.00
>1	120	23.1%	120	23.1%	0.00
Clozapine	18	3.5%	18	3.5%	0.00
Prior year number of medication classes dispensed					
0-1	179	34.5%	161	31.0%	0.07
2-5	186	35.8%	177	34.1%	0.04
>5	154	29.7%	181	34.9%	0.11

Prior year medication use					
Mood stabilizer	62	11.9%	51	9.8%	0.07
Antidepressant	151	29.1%	184	35.5%	0.14
Anxiolytic	152	29.3%	163	31.4%	0.05
Sedative-hypnotic	86	16.6%	90	17.3%	0.02
Anticonvulsant	28	5.4%	30	5.8%	0.02
Psychostimulant	9	1.7%	19	3.7%	0.12
Anticholinergic	93	17.9%	81	15.6%	0.06
Opioid	80	15.4%	112	21.6%	0.16
Opioid agonist therapy	S	S	6	1.2%	0.09
Smoking cessation aid	15	2.9%	10	1.9%	0.06
Alcohol use disorder drug	S	S	S	S	0.04
Dementia drug	S	S	S	S	0.08
Antidiabetic drug	43	8.3%	34	6.6%	0.01
Antihyperlipidemic drug	34	6.6%	35	6.7%	0.07
Comorbidities					
Mood or anxiety disorder	381	73.4%	410	79.0%	0.13
Personality disorder	133	25.6%	135	26.0%	0.01
Substance use disorder	278	53.6%	260	50.1%	0.07
Dementia	64	12.3%	57	11.0%	0.04
Autism spectrum disorder	12	2.3%	15	2.9%	0.04
Intellectual disability/ developmental disorder	51	9.8%	49	9.4%	0.01
ADHD	65	12.5%	67	12.9%	0.01

Suicide attempt	45	8.7%	51	9.8%	0.04
Prior year hospitalizations					
0	180	34.7%	167	32.2%	0.05
1-2	303	58.4%	307	59.2%	0.02
>2	36	6.9%	45	8.7%	0.06
Prior year physician visits					
0-2	23	4.4%	32	6.2%	0.08
3-5	52	10.0%	48	9.2%	0.03
>5	444	85.5%	439	84.6%	0.03
Incidents where accused of a crime					
0	361	69.6%	389	75.0%	0.12
1-2	69	13.3%	61	11.8%	0.05
>2	89	17.1%	69	13.3%	0.11
Incidents where victim of a crime					
0	495	95.4%	497	95.8%	0.02
>0	24	4.6%	22	4.2%	0.02

ADHD=attention-deficit hyperactivity disorder; FGA=first-generation antipsychotic; FG-LAIA=first-generation long-acting injectable antipsychotic; LAI=long-acting injectable; S=suppressed due to count <6; SD=standard deviation; SGA=second-generation antipsychotic; SG-LAIA=second-generation long-acting injectable antipsychotic

Supplementary Table 5. Antipsychotics used as monotherapy by cohort members prior to and upon cohort entry.

	Matched cohort				
	SG-LAIA New Users, n=519		Antipsychotic Users, n=519		Standard-ized
Characteristics	n or mean	% or SD	n or mean	% or SD	Difference
Prevalent antipsychotic used prior to cohort entry†					
SGA	360	69.4%	359	69.2%	0.01
Aripiprazole	36	6.9%	25	4.8%	0.10
Brexpiprazole	0	0.0%	0	0.0%	0.00
Clozapine	14	2.7%	8	1.5%	0.09
Lurasidone	S	S	S	S	0.07
Olanzapine	110	21.2%	101	19.5%	0.05
Paliperidone	S	S	S	S	0.00
Quetiapine	69	13.3%	86	16.6%	0.11
Risperidone	128	24.7%	138	26.6%	0.05
Ziprasidone	S	S	0	0.0%	0.07
FGA	41	7.9%	42	8.1%	0.01
Chlorpromazine	S	S	S	S	0.10
Flupentixol	0	0.0%	0	0.0%	0.00
Flupentixol LAI	9	1.7%	7	1.3%	0.04
Fluphenazine	0	0.0%	0	0.0%	0.00
Fluphenazine LAI	7	1.3%	S	S	0.15
Haloperidol	S	S	9	1.7%	0.08
Haloperidol LAI	6	1.2%	9	1.7%	0.06

Loxapine	S	S	S	S	0.00
Methotrimeprazine	S	S	S	S	0.00
Perphenazine	S	S	S	S	0.07
Pimozide	S	S	S	S	0.07
Pipotiazine palmitate LAI	0	0.0%	0	0.0%	0.00
Thiothixene	0	0.0%	0	0.0%	0.00
Trifluoperazine	S	S	0	0.0%	0.10
Zuclopenthixol	S	2.0%	S	S	0.04
Zuclopenthixol LAI	S	S	S	S	0.00
None	118	22.7%	118	22.7%	0.00
Antipsychotic dispensed on t_0					
SG-LAIA					
Aripiprazole LAI	58	11.2%	-	-	-
Paliperidone LAI‡	198	38.2%	-	-	-
Risperidone LAI	263	50.7%	-	-	-
SGA	-	-	463	89.2%	-
Aripiprazole	-	-	33	6.4%	-
Brexpiprazole	-	-	S	S	-
Clozapine	-	-	S	S	-
Lurasidone	-	-	S	S	-
Olanzapine	-	-	136	26.2%	-
Paliperidone	-	-	S	S	-
Quetiapine	-	-	105	20.2%	-

Risperidone	-	-	179	34.5%	-
Ziprasidone	-	-	S	S	-
FGA	-	-	56	10.8%	-
Chlorpromazine	-	-	S	S	-
Flupentixol	-	-	0	0.0%	-
Flupentixol LAI	-	-	9	1.7%	-
Fluphenazine	-	-	S	S	-
Fluphenazine LAI	-	-	S	S	-
Haloperidol	-	-	13	2.5%	-
Haloperidol LAI	-	-	11	2.1%	-
Loxapine	-	-	S	S	-
Methotrimeprazine	-	-	S	S	-
Perphenazine	-	-	0	0.0%	-
Pimozide	-	-	S	S	-
Trifluoperazine	-	-	0	0.0%	-
Zuclopenthixol	-	-	S	S	-
Zuclopenthixol LAI	-	-	S	S	-
Total FG-LAIA	-	-	27	5.2%	-

†If multiple prior antipsychotics were used, the antipsychotic with longest duration of continuous use was selected
‡Includes once-monthly and 3-monthly formulations of paliperidone
FGA=first-generation antipsychotic; FG-LAIA=first-generation long-acting injectable antipsychotic; LAI=long-acting injectable; S=suppressed due to count <6; SD=standard deviation; SGA=second-generation antipsychotic; SG-LAIA=second-generation long-acting injectable antipsychotic; t_0=cohort entry date

Supplementary Table 6. Sensitivity analysis: Antipsychotics used as monotherapy by cohort members prior to and upon cohort entry, with inclusion of previous antipsychotic in time-conditional propensity score.

	Matched cohort				
	SG-LAIA New Users, n=1,164		Antipsychotic Users, n=1,164		Standardized
Characteristics	n or mean	% or SD	n or mean	% or SD	Difference
Prevalent antipsychotic used prior to cohort entry†					
SGA	864	74.2%	848	72.9%	0.05
Aripiprazole	64	5.5%	57	4.9%	0.03
Brexpiprazole	0	0.0%	0	0.0%	0.00
Clozapine	44	3.8%	36	3.1%	0.04
Lurasidone	S	S	S	S	0.02
Olanzapine	257	22.1%	237	20.4%	0.05
Paliperidone	S	S	S	S	0.00
Quetiapine	205	17.6%	220	18.9%	0.04
Risperidone	284	24.4%	294	25.3%	0.02
Ziprasidone	7	0.6%	0	0.0%	0.12
FGA	113	9.7%	129	11.1%	0.05
Chlorpromazine	S	S	10	0.9%	0.06
Flupentixol	S	S	S	S	0.05
Flupentixol LAI	17	1.5%	15	1.3%	0.02
Fluphenazine	0	0.0%	S	S	0.05
Fluphenazine LAI	22	1.9%	18	1.5%	0.03
Haloperidol	24	2.1%	27	2.3%	0.02
Haloperidol LAI	12	1.0%	14	1.2%	0.02

Loxapine	7	0.6%	8	0.7%	0.01
Methotrimeprazine	9	0.8%	11	0.9%	0.02
Perphenazine	S	S	S	S	0.05
Pimozide	S	S	S	S	0.02
Pipotiazine palmitate LAI	0	0.0%	0	0.0%	0.00
Thiothixene	0	0.0%	0	0.0%	0.00
Trifluoperazine	S	S	S	S	0.02
Zuclopenthixol	S	S	S	S	0.06
Zuclopenthixol LAI	8	0.7%	7	0.6%	0.01
None	187	16.1%	187	16.1%	0.00
Antipsychotic dispensed on t_0					
SG-LAIA					
Aripiprazole LAI	128	11.0%	-	-	-
Paliperidone LAI‡	445	38.2%	-	-	-
Risperidone LAI	591	50.8%	-	-	-
SGA	-	-	1013	87.0%	-
Aripiprazole	-	-	71	6.1%	-
Brexpiprazole	-	-	S	S	-
Clozapine	-	-	36	3.1%	-
Lurasidone	-	-	S	S	-
Olanzapine	-	-	288	24.7%	-
Paliperidone	-	-	S	S	-
Quetiapine	-	-	253	21.7%	-

Risperidone	-	-	360	30.9%	-
Ziprasidone	-	-	0	0.0%	-
FGA	-	-	151	13.0%	-
Chlorpromazine	-	-	11	0.9%	-
Flupentixol	-	-	S	S	-
Flupentixol LAI	-	-	17	1.5%	-
Fluphenazine	-	-	S	S	-
Fluphenazine LAI	-	-	24	2.1%	-
Haloperidol	-	-	37	3.2%	-
Haloperidol LAI	-	-	17	1.5%	-
Loxapine	-	-	9	0.8%	-
Methotrimeprazine	-	-	13	1.1%	-
Perphenazine	-	-	0	0.0%	-
Pimozide	-	-	S	S	-
Trifluoperazine	-	-	S	S	-
Zuclopenthixol	-	-	S	S	-
Zuclopenthixol LAI	-	-	8	0.7%	-
Total FG-LAIA	-	-	66	5.7%	-

†If multiple prior antipsychotics were used, the antipsychotic with longest duration of continuous use was selected
‡Includes once-monthly and 3-monthly formulations of paliperidone
FGA=first-generation antipsychotic; FG-LAIA=first-generation long-acting injectable antipsychotic; LAI=long-acting injectable; S=suppressed due to count <6; SD=standard deviation; SGA=second-generation antipsychotic; SG-LAIA=second-generation long-acting injectable antipsychotic; t_0=cohort entry date

Supplementary Table 7. Sensitivity analysis: Association between second-generation long-acting injectable antipsychotics versus oral antipsychotics and treatment failure, psychiatric hospitalization, incarceration, treatment discontinuation and all-cause mortality with inclusion of previous antipsychotic in time-conditional propensity score.

	Number of events	Person years	Crude incidence rate per 100 person years	Crude Hazard Ratio (95% CI)		Adjusted Hazard Ratio* (95% CI)	
Treatment failure							
SG-LAIA New Users	905	1,491	60.7	1.15	(1.11-1.19)	1.05	(0.97-1.13)
Matched Antipsychotic Users	787	1,717	45.8	1.00	(Reference)	1.00	(Reference)
Psychiatric hospitalization							
SG-LAIA New Users	572	2,792	20.5	1.21	(1.14-1.29)	1.35	(1.21-1.51)
Matched Antipsychotic Users	471	2,890	16.3	1.00	(Reference)	1.00	(Reference)
Incarceration							
SG-LAIA New Users	173	4,396	3.9	1.18	(1.02-1.36)	0.99	(0.75-1.30)
Matched Antipsychotic Users	147	3,904	3.8	1.00	(Reference)	1.00	(Reference)
Treatment discontinuation							
SG-LAIA New Users	797	2,148	37.1	1.21	(1.16-1.26)	1.01	(0.93-1.10)
Matched Antipsychotic Users	658	2,197	29.9	1.00	(Reference)	1.00	(Reference)
All-cause mortality							
SG-LAIA New Users	95	5,184	1.8	0.99	(0.82-1.19)	1.10	(0.77-1.57)
Matched Antipsychotic Users	96	4,383	2.2	1.00	(Reference)	1.00	(Reference)

*Adjusted for time-varying use of additional antipsychotic medications in each 3-month period of follow-up time, and baseline variables age, sex, time since psychotic disorder diagnosis, decile of time-conditional propensity score, prior year hospital admissions, being accused of a crime, diagnosis of personality disorder, substance use disorder, mood/anxiety disorder

SG-LAIA=second-generation long-acting injectable antipsychotic

Supplementary Figure 1. Outcome comparison in a cohort with psychotic disorder versus a cohort with schizophrenia.

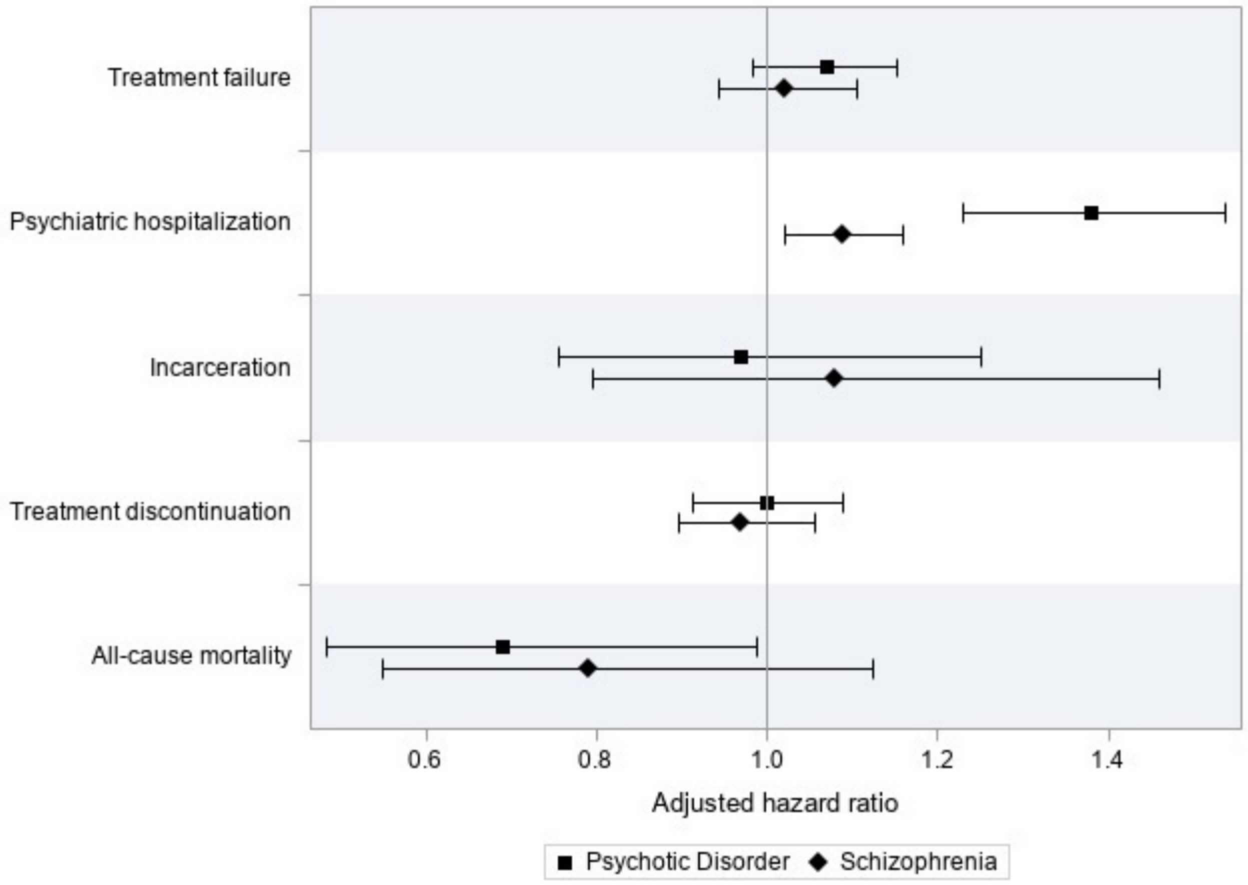

Supplementary Figure 2. Outcome comparison during monotherapy in a cohort with psychotic disorder versus a cohort with schizophrenia.

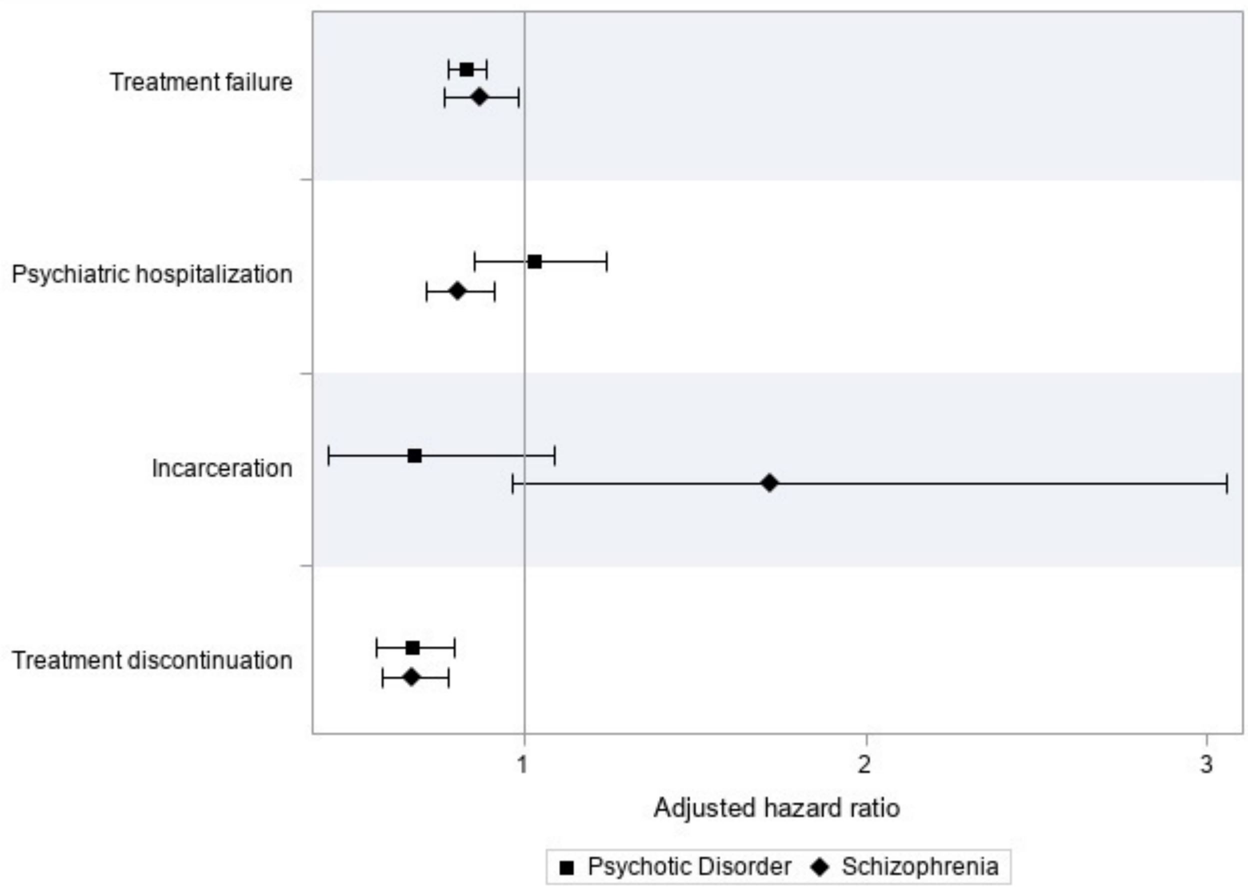

Supplement: Supplementary file 1 [file DataSheet1.pdf]
